# Rational Design of Biological Crystals with Enhanced Physical Properties by Hydrogen Bonding Interactions

**DOI:** 10.34133/research.0046

**Published:** 2023-02-24

**Authors:** Hui Yuan, Bin Xue, Dingyi Yang, Sigal Rencus-Lazar, Yi Cao, Ehud Gazit, Dan Tan, Rusen Yang

**Affiliations:** ^1^ School of Advanced Materials and Nanotechnology, Xidian University, Xi’an 710126, China.; ^2^ The Shmunis School of Biomedicine and Cancer Research, George S. Wise Faculty of Life Sciences, Department of Materials Science and Engineering, The Iby and Aladar Fleischman Faculty of Engineering, Tel Aviv University, Tel Aviv 6997801, Israel.; ^3^ National Laboratory of Solid State Microstructure, Department of Physics, Nanjing University, Nanjing 210093, Jiangsu, China.

## Abstract

Hydrogen bonds are non-covalent interactions and essential for assembling supermolecules into ordered structures in biological systems, endowing crystals with fascinating physical properties, and inspiring the construction of eco-friendly electromechanical devices. However, the interplay between hydrogen bonding and the physical properties is not fully understood at the molecular level. Herein, we demonstrate that the physical property of biological crystals with double-layer structures could be enhanced by rationally controlling hydrogen bonding interactions between amino and carboxyl groups. Different hydrogen bonding interactions result in various thermal, mechanical, electronic, and piezoelectric properties. In particular, the weak interaction between O and H atoms contributes to low mechanical strength that permits important ion displacement under stress, giving rise to a strong piezoelectric response. This study not only reveals the correlation between the hydrogen bonding and physical properties in double-layer structures of biological crystals but also demonstrates the potential of these crystals as functional biomaterials for high-performance energy-harvesting devices. Theoretical calculations and experimental verifications in this work provide new insights into the rational design of biomaterials with desirable physical properties for bioelectrical devices by modulating intermolecular interactions.

## Introduction

Biomaterials with fascinating biocompatibility, bio-functionalization, and environmental friendliness are highly desirable for biological engineering, allowing to complement the application of inorganic and polymer-based materials [1–4]. Notably, a broad range of biomaterials including proteins, peptides, and amino acids demonstrate stable structures with important mechanical and piezoelectric properties, making them promising candidates to be engineered and exploited in energy harvesting, strain sensors, actuators, bioimaging, and tissue engineering applications [5–10]. Despite the advancements in developing functional biomaterials for various applications, the physical property of biomaterials induced by supramolecular packing are not fully understood at the molecular level [7,11]. In biological systems, the physical properties were found to be influenced by functional groups and non-covalent interactions, which are distinguished from traditional inorganic materials [12–15]. A deep understanding of the physical properties of supramolecular biomaterials is critical for the development of high-performance and ultrasensitive transient electronic devices.

Many theoretical and experimental studies have been conducted to understand and improve the physical properties of biomaterials [16]. For example, the mechanical property of biological crystals was founded to be crystal face-independent, with the highest mechanical property measured perpendicular to the primary direction of hydrogen bonding networks [15]. Aromatic interactions in peptides predominantly resulted in high thermostable structures [17]. The addition of a hydroxyl group to helical structures of diphenylalanine-derived tripeptides showed enhanced crystal polarizability under stress, resulting in higher piezoelectric properties [7]. Moreover, adjusting the guest–host interaction of a peptide-based metal–organic framework yielded a significant enhancement in the electromechanical response because of the lower symmetry of the crystal [11]. It is worth noting that the physical properties of crystals are closely related to their hydrogen bonding interactions, one of the most crucial and essential non-covalent interactions that significantly affect molecular packing. However, the effect of the hydrogen bonding interactions on physical properties in biomaterials is still not fully understood, hindering the rational design of biomaterials with desired properties.

Herein, we demonstrate the enhanced physical property of biological crystals through rationally modulating their hydrogen bonding interactions. Various side group amino acids composed of valine, leucine, and methionine were packed into a non-centrosymmetric *P*2_1_ space group with a similar hydrogen bonding arrangement yet different interaction. The varying hydrogen bonding interactions resulted in different physical properties including thermal, mechanical, electronic, and piezoelectric properties, according to atomic force microscopy (AFM) measurements and density functional theory (DFT) calculations. Notably, the leucine crystals with the weakest hydrogen bonding interaction exhibited the lowest Young’s modulus and the highest piezoelectric coefficient (*d*_16_ = 42.6 pm V^−1^), followed by methionine crystals with a strong net polarization along the *b* axis that exhibited a high piezoelectric response (*d*_22_ = 37.6 pm V^−1^). The thermostable structure and significant piezoelectric response of methionine crystals allowed the fabrication of environment-friendly and stable nanogenerators that can generate a high output voltage of 1.63 V under an applied force of 42 N.

## Results and Discussions

**Fig. 1.**
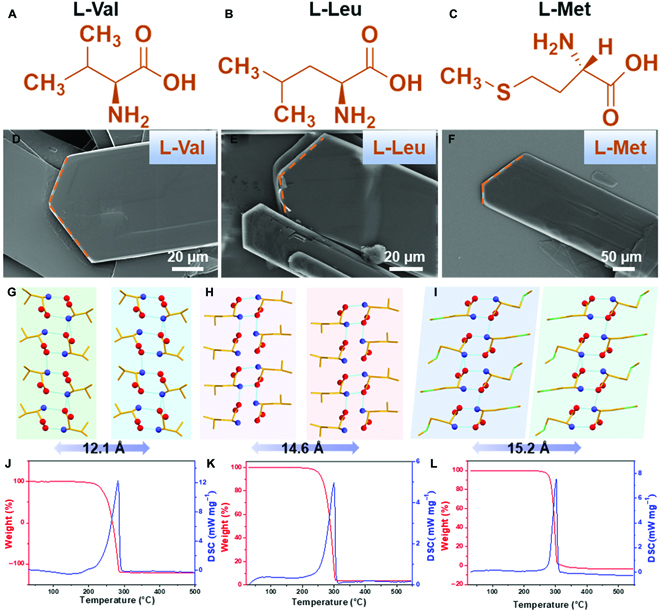
The morphology, structure, and thermostability of the examined amino acid crystals. (A to C) Chemical structures of (A) valine, (B) leucine, and (C) methionine. (D to F) SEM images of (D) valine, (E) leucine, and (F) methionine. (G to I) Crystal structures of (G) valine, (H) leucine, and (I) methionine. Color code: orange, C; blue, N; red, O; light green, S. (J to L) Thermal stability measurements of (J) valine, (K) leucine, and (L) methionine.

Amino acid groups composed of valine, leucine, and methionine were chosen on the basis of their similar chemical structures with various lengths and atoms to achieve supramolecular packing with different hydrogen bonding interactions (Fig. 1A to C). These amino acid molecules self-assembled into thin-sheet crystals with similar morphologies in water and MeOH mixed solutions (Fig. S1A), and the leucine sheets were slightly thicker than the other 2 crystals (Fig. 1D to F). Because of the different solubility of the amino acids in solvents of various polarizations, solvents may affect their assembly processes [11]. We observed that the growth speed and density of amino acid crystals in various solvents including ethanol, isopropanol, MeOH, and acetonitrile were different yet showed the same variation trend for the different amino acids (Fig. S1B to D), owing to the various thermodynamics and the kinetics of the crystallization process [11]. Similar crystal morphologies were observed in ethanol/water and isopropanol/water mixed solutions (Figs. S2 to S4). Crystals grown in a MeOH/water mixture presented angular sheet morphologies with larger shapes compared with crystals grown in other solutions, probably owing to the higher solubility-induced lower nucleation kinetics and higher growth kinetics (Figs. S2 to S4). However, altering the solvent into acetonitrile yielded a mass of small crystals because of the accelerated nucleation and limited growth process (Figs. S2 to S4). Thus, various morphologies of amino acid crystals were formed by controlling their nucleation and growth processes.

X-ray diffraction (XRD) measurements and simulation results demonstrated that the amino acids self-assembled into non-centrosymmetric crystals with a space group *P*2_1_ in various solvents (Figs. S5 to S7), potentially having piezoelectric properties [18–20]. The diffraction peaks of crystals assembled from different solvents did not show an obvious change in terms of the peak position, indicating the formation of pure and same polymorphs (Figs. S5 to S7). These amino acid molecules are packed into layer structures by forming head-to-tail hydrogen bonds (O22…N11, 3.07 Å and N11…O12, 2.92 Å for valine; N1…O3, 2.79 Å for leucine; and N2…O1, 2.98 Å and N2…O3, 2.86 Å for methionine) between amino and carboxyl groups. The ordered hydrogen bonding networks (O21…N21, 2.87 Å and O22…N11, 2.77 Å for valine; N2…O1, 2.76 Å and O3…N1, 2.81 Å for leucine; and O2…N1, 2.84 Å and O1…N2, 2.78 Å for methionine) allowed amino acid molecules to align along the *a* or *c* axis, forming double-layer structures (Fig. S8) [19,21,22]. The layer structure further extended to form (010) crystal planes. Although these hydrogen bonding networks aligned in a similar direction, the various side chains of the tested amino acids yielded different layer space distances. Following their different lengths of molecular chains, the interlayer distance of layer space for the 3 amino acids is in the order of methionine (15.2 Å), leucine (14.6 Å), and valine (12.3 Å) (Fig. 1G to I), indicating the wider interplanar distance in the methionine crystal [23].

The strong hydrogen bonding network enables stable molecular packing, as verified by thermogravimetric analysis (TGA) and differential scanning calorimetry analyses. As illustrated in Fig. 1J to L, the TGA curves of these amino acid crystals present a slight weight loss before 200 °C, indicating good stability without polymorphic transformation. Among the samples, methionine crystals demonstrated the best thermostability and were stable up to ~220 °C.

**Fig. 2.**
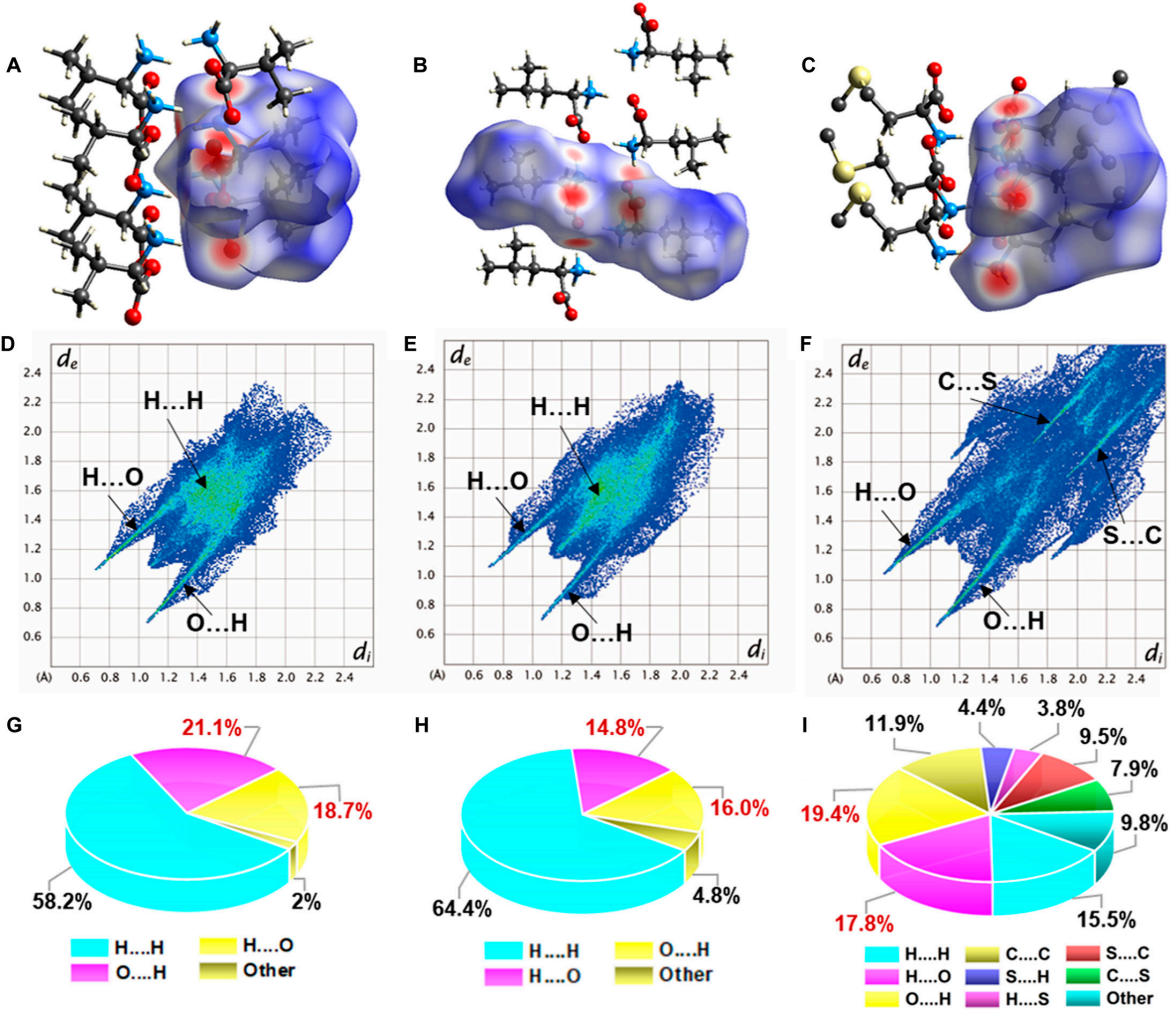
Molecular interactions of amino acids. Hershfield surfaces of (A) valine, (B) leucine, and (C) methionine mapped over *d*_norm_. (D to F) 2D fingerprint plots of (D) valine, (E) leucine, and (F) methionine. (G to I) Relative contribution of the specific interactions to the Hirshfeld surface for (G) valine, (H) leucine, and (I) methionine.

Even though the molecular packing was evident in crystal structures, hydrogen bonding interactions cannot be accurately understood by simply measuring their bond lengths and directions. Hirshfeld surface analysis is an efficient tool to analyze interactions between atoms in crystal structures by partitioning the crystal space occupied by molecules into nonoverlapping and interlocking molecular volumes based on their crystal electron density [24,25]. The Hirshfeld surfaces of valine, leucine, and methionine crystals were mapped with the parameter *d*_norm_ (Fig. 2A to C), and 2 molecules were comprised inside the Hirshfeld surface. Red spots on the surface near O atoms represent the strong interaction between O and H atoms, and the blue area indicates weak interactions contributed by interatomic contact with a distance beyond the van der Waals distance. The interatomic contact in valine, leucine, and methionine crystals was quantified by plotting 2-dimensional (2D) fingerprint plots (Fig. 2D to F), and the relative contribution of each interatomic contact to the Hirshfeld surface was displayed in 3 pie charts (Fig. 2G to I). All amino acid crystals mapping on the Hirshfeld surface demonstrated high contact percentage between O and H atoms, yet the difference was found in the contribution of hydrogen bonding interactions to all the interatomic interactions. The H...O and O...H contracts in valine crystals displayed the highest value of 39.8%, followed by methionine crystals (37.2%) and leucine crystals (30.8%). As a result, these amino acid crystals displayed hydrogen bonding interactions between amino and carboxyl groups in the order from high to low as valine, methionine, and leucine. Notably, weak interactions between C and S atoms (12.3%) and H and S atoms (8.2%) were comprised in methionine crystals, probably contributing to electric properties. The detailed hydrogen bonding energy needs to be further calculated by Kohn–Sham DFT methods [26]. Here, we mainly focus on the contribution of hydrogen bonding to the whole crystal structure.

**Fig. 3.**
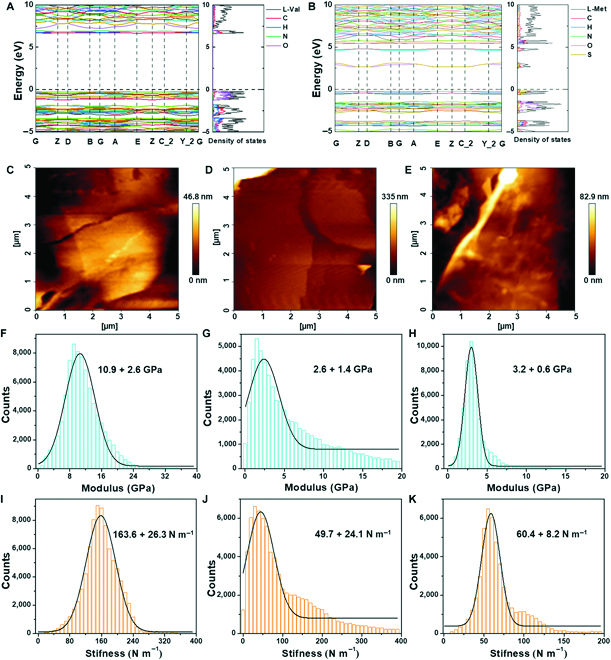
The influence of hydrogen interactions on the electrical and mechanical property of amino acids. (A and B) Band structures and density of states of (A) valine and (B) methionine. (C to E) AFM topography images of (C) valine, (D) leucine, and (E) methionine. (F to H) Young’s modulus of (F) valine, (G) leucine, and (H) methionine. (I to K) Point stiffness of (I) valine, (J) leucine, and (K) methionine.

Next, the influence of interatomic interactions on electric band structures and density of state (DOS) properties was explored by DFT calculations. The molecular packing of the crystals in different directions as shown in Fig. S9 displays a similar amino acid arrangement, indicating that the difference in calculated electronic band structures and the corresponding DOS primarily resulted from the various interatomic interactions. The indirect bandgap of valine and leucine crystals were calculated to be ~7.1 and ~6.28 eV (Fig. 3A and Fig. S10), respectively, indicating insulating properties. However, the indirect bandgap of methionine crystals decreased to ~2.9 eV because of the interactions between C and S atoms (Fig. 3B), indicating its potential for the design of high-performance electronic devices based on biomaterials. The varying bandgap structures resulting from the interaction of atoms were revealed in detail via partial DOS of s-states, p-states, and d-states of atoms (Figs. S11 to S13). The H, C, and S atoms in methionine crystals contributed to the energy region close to the band edge position (Fig. S13), while the H and C atoms in valine and leucine crystals showed wide energy gaps (Figs. S11 and S12). The result suggested that the smaller bandgap in methionine crystals mainly emanated from the S–C bond, which resulted in higher energy in the highest occupied molecular orbital (HOMO) compared to valine and leucine (Fig. S14).

We further explore the correlation between hydrogen bonding interactions and mechanical properties. The Young’s modulus of valine, leucine, and methionine crystals along the thickness direction was measured by AFM nanoindentation, with a tip scanning the smooth surface of the crystals with a size of 5 × 5 μm at different positions (Fig. 3C to E). Typically, the cantilever was forced to press the surface of the crystals and retracted at a constant speed of 30 μm s^−1^, during which force–displacement traces were recorded (Fig. S15). The Young’s modulus of the crystals at different positions could be determined by fitting the retracting traces using the Hertz model. The valine crystals showed the highest Young’s modulus of 10.9 ± 2.6 GPa, while the leucine and methionine crystals demonstrated lower Young’s modulus values of 2.6 ± 1.4 and 3.2 ± 0.6 GPa, respectively (Fig. 3F to H). On the basis of force–displacement traces, the corresponding point stiffnesses of valine, leucine, and methionine crystals were calculated to be 163.6 ± 26.3, 49.7 ± 24.1, and 60.4 ± 8.2 N m^−1^, respectively (Fig. 3I to K). These high values relative to other biomaterials could potentially allow practical applications. Notably, the strong hydrogen bonding interactions of valine crystals contributed to the high mechanical stiffness, where the strong interactions between O and H atoms may resist the deformation of crystals, playing an important role in structural stability.

**Fig. 4.**
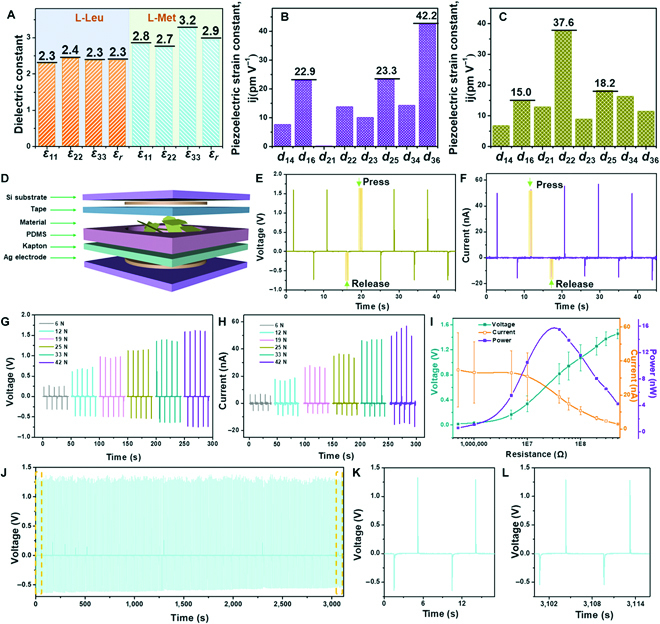
The influence of hydrogen interactions on the piezoelectric property of amino acid crystals. (A) The calculated dielectric constant of leucine and methionine crystals. (B) The calculated piezoelectric constant of leucine crystals. (C) The calculated piezoelectric constant of methionine crystals. (D) The schematic diagram of nanogenerators based on methionine. (E) Open-circuit voltage and (F) short-circuit current from methionine-based nanogenerators under an applied force of 42 N. (G) Open-circuit voltage and (H) short-circuit current from methionine-based nanogenerators with various forces. (I) Output voltage, current, and power of methionine-based nanogenerators with various resistance loads. (J to L) Output voltage of methionine-based nanogenerators over time. (K and L) The output voltage of methionine-based nanogenerators in the (K) initial and (L) last round during the continuous compression-relaxation cycles presented in (J).

The mechanical properties of these amino acid crystals have been shown to be affected by hydrogen bonding interactions, potentially affecting their piezoelectric properties. To verify this hypothesis, DFT calculations were carried out to examine their dielectric, elastic, and piezoelectric constants. The total dipole moment in a dielectric material can be reflected by the relative permittivity, which is related to the piezoelectric response. The average dielectric constants ɛ_r_ of valine, leucine, and methionine crystals were calculated to be 2.4, 2.3, and 2.9, respectively, and these low values allowed for high piezoelectricity (Fig. 4a) [5,11,27]. The piezoelectric constant tensors composed of 1 longitudinal, 2 transverse, and 5 shear nonzero piezoelectric coefficients were calculated on the basis of the various directions of elastic constants (Fig. 4B and C and Tables S1 and S2). Methionine crystals demonstrated high piezoelectric responses, with a high longitudinal piezoelectric constant *d*_22_ = 37.6 pm V^−1^, which attributed to the loose molecular arrangement and low stiffness of the crystal (Fig. 4C and Table S2). Interestingly, leucine crystals with weaker hydrogen bonding interactions demonstrated a maximal shear piezoelectric constant *d*_36_ = 42.2 pm V^−1^ (Fig. 4B and Table S1), higher than many amino acid, dipeptide, and tripeptide crystals (Fig. S16) [5,7,28–31]. Among the 3 amino acids, valine crystals with the highest mechanical property showed the lowest piezoelectric constant (*d*_22_ = 12.3 pm V^−1^) [27]. The high piezoelectric response of the leucine crystals can be attributed to the weak hydrogen bonding interaction-induced low stiffness that promoted ion displacement under mechanical forces, leading to a large electric dipole moment. Moreover, the arrangement direction and distance of hydrogen bonding may influence the electric dipoles, which further affect piezoelectric properties [14,32]. These calculated results reveal that the piezoelectric response of the tested biological crystals is strongly correlated with the hydrogen bonding interactions and that the weak interaction enables high electromechanical properties.

The high piezoelectric response and high-temperature stability (Fig. S17) may imply methionine crystals with excellent high-temperature piezoelectric properties, which were further evaluated by piezoresponse force microscopy (PFM) with a heating system. At room temperature, the methionine crystals demonstrated the effective piezoelectric constant ranging from 2.3 to 18.0 pm V^−1^ along the thickness direction and showed a maximal tested piezoelectric constant of 18.0 pm V^−1^ (Fig. S18). In situ high-temperature piezoelectric measurements showed that the piezoelectric response of the methionine crystals slightly increased upon increasing the temperature between 50 and 125 °C, indicating good thermostable piezoelectricity (Fig. S19A). The slight increase in piezoelectricity may be explained by the heat energy on the surface of the crystals at high temperatures. To eliminate the influence of heat energy on the movement of tips at high temperatures, we measured the piezoelectric constant of the cooled crystals after annealing at 60, 80, 100, 120, and 150 °C for 0.5 h. The crystal showed an effective piezoelectric coefficient of 14.9 pm V^−1^ after annealing at 60 °C, which was maintained up to 14.26 pm V^−1^ after annealing at 120 °C (Fig. S19B). The negligible change in piezoelectric constants at an elevated temperature confirms that these materials have very good thermostability (Fig. S19B). Their thermostable piezoelectric response is better than phenylalanine, β-glycine, and diphenylalanine crystals [5,33]. Even though the β-glycine crystal demonstrated a higher shear piezoelectric constant of 178 pm V^−1^, the crystal structure was reported to be metastable and easily transformed into stable α- and γ-polymorph in the natural environment, with a drastically reduced piezoelectric response [5]. Thus, the methionine crystals with good piezoelectricity and stable structures at high temperatures may be suitable for micromechanical applications.

To test their applicative potential, methionine crystals were exploited in emerging nanogenerators for energy harvesting. The crystals filled with polydimethylsiloxane (PDMS) were sandwiched between 2 Ag electrodes (Fig. 4D). Two Si wafers were utilized as top and bottom substrates to support the device (Fig. 4D). When a mechanical force is applied to the device, polarization charges are created by the slight separation of positive and negative ions in the piezoelectric methionine crystals, thereby driving the flow of external free electrons (Fig. S20). The methionine-based nanogenerator produced an open-circuit voltage of 1.62 V and a short-circuit current of 55.1 nA under an applied force of 42 N (Fig. 4E and F). The output signals were force-dependent, and the output voltage increased from 0.28 to 1.62 V with the increased forces from 6 to 42 N (Fig. 4G), showing a linear trend. Correspondingly, the output current increased linearly from 6.3 to 55.1 nA as a function of applied forces increasing from 6 to 42 N (Fig. 4H), indicating high piezoelectric responses. The output voltage and current signals can be reversed in switching polarity tests (Fig. S20), indicating that the measured signals are truly generated by the piezoelectric nanogenerator device [34,35]. A reversed voltage of 1.54 V and a reversed current of 55.2 nA were obtained under an applied force of 42 N, and the output signal also presented a linear increase with forces (Fig. S21). As a control experiment, valine crystals were fabricated into nanogenerator devices. The device generated an open-circuit voltage of ~0.3 V and a short-circuit current of ~15.2 nA under a mechanical force of 42 N (Fig. S22), ~5.4 and ~3.6 times smaller than that of devices based on methionine with a similar force, respectively. The significantly enhanced output of the methionine-based nanogenerator can be attributed to the optimized piezoelectric coefficient by engineering weak hydrogen bonding interactions of biological crystals, highlighting the dramatic effect of molecular engineering.

When the nanogenerator was connected to various external resistive loads ranging from 0.5 to 500 MΩ, the output voltage increased with resistances and the output current decreased with resistances. The nanogenerator produced a maximal power of 15.4 nW (31.4 nW cm^−2^) with a resistance of 40 MΩ (Fig. 4I). The methionine crystal with stable structures and mechanical properties enables the device to bear cyclic impact forces and produce a stable power supply. As illustrated in Fig. 4J to L, the methionine-based nanogenerator generates stable voltage signals without degradation for more than 3110 s. Hence, the methionine crystals in our work are proven to be stable and highly efficient piezoelectric biomaterials that are promising candidates for eco-friendly electromechanical systems.

## Conclusion

In this study, we demonstrated a physical property-enhanced strategy by taking advantage of the hydrogen bonding networks of double-layer amino acid crystals with their molecular side chains. The diverse interatomic interactions yield a significant difference in their energy band structures. The interactions between S and C atoms dramatically decrease the bandgap of biological crystals and enable their potential application in biomaterial-based electronic devices. The strong interactions strengthen the mechanical properties and robustness of the biological crystals, and the stable hydrogen network improves their resistance to mechanical force-induced damage. Notably, the biological crystals were found to be piezoelectric, with a maximal shear piezoelectric coefficient of *d*_36_ = 42.2 pm V^−1^ in leucine crystals and a maximal longitudinal piezoelectric coefficient of *d*_22_ = 37.6 pm V^−1^ in methionine crystals, due to their flexibility properties caused by weak interactions and large ion replacement under the applied stress. The methionine crystals with thermostable structures and piezoelectricity were employed in a nanogenerator that generated a maximal output voltage of 1.62 V, a maximal output current of 55.1 nA, and a maximal power of 15.4 nW, demonstrating their potential for eco-friendly energy-harvesting devices.

## Materials and Methods

### Crystal preparation

L-valine, L-leucine, and L-methionine amino acids (China National Medicines Corporation Ltd., China) were dissolved in deionized water at concentrations of 20, 30, and 40 mg ml^−1^, respectively, forming clear solutions. Six milliliters of ethanol, isopropanol, MeOH, and acetonitrile solvents were slowly added to the above solutions. The crystals precipitated in a few minutes and reached the maximal size after 1 week. The crystals were washed with ethanol several times and dried at 60 °C for 6 h to obtain pure crystals.

### X-ray diffraction

The XRD patterns of amino acid crystals self-assembled under different conditions were collected using a D8 DISCOVER diffractometer with Cu Kα radiation. A quartz zero-background sample holder was used for all powder samples. All the samples were scanned with an angle range of 5 to 40° and a step of 0.02°.

### Scanning electron microscopy and thermogravimetric analysis

Amino acid crystals were uniformly dispersed in an ethanol solution and dropped on aluminum foils. After drying, the samples were pasted on a holder using conductive tapes. In order to increase the conductivity of the samples, an Au film was sputtered on the surface of the samples before observation. We utilized an Apreo+HiVac field emission scanning electron microscope to observe the morphology of amino acid samples, with an operating voltage of 2 kV and an operating current of 25 pA. The thermostability of the samples was characterized using a STA+449F5 simultaneous thermal analyzer, with a heating ratio of 10 °C min^−1^ in the air.

### Young’s modulus measurements

AFM nanoindentation experiments were performed using a commercial AFM (JPK, Nanowizard IV, Berlin, Germany). The crystals were spread on a mica substrate and then blown with nitrogen to remove the loose samples. Typically, the nanoindentation was performed on a flat area (scan area: 5 × 5 μm) at the QI mode (conditions: pixels: 60 × 60; *Z* length: 0.1 μm; extend and retract speed: 30 μm s^−1^; *Z* resolution: 80,000 Hz; maximum loading force: 1,200 nN), and RTESPA-525 cantilevers (Bruker Company; half-open angle of the pyramidal face of θ: <10°, tip radius: ~20 nm, and spring constant: ~200 N m^−1^) were used in all experiments. The cantilever was extended to the surface of the crystal and retracted. The force and displacement during the process were recorded. Young’s modulus of the crystals was calculated by fitting the extending curve with the Hertz model (Eq. 1).

F=43E1−ν2Rδ32 (1)

where *F* corresponds to the force, δ corresponds to the depth of the crystal pressed by the cantilever tip, *R* is the radius of the tip, *E* is the Young’s modulus of the crystals, and ν is the Poisson ratio (ν = 0.3). The point stiffness was determined as the normal force divided by the deformation of the sample and calculated from the force–displacement curves after deducting the deformation of the cantilever. For each sample, more than 6 locations were randomly selected to perform the nanoindentation. All data were analyzed using the JPK data processing 7.0.46 software (JPK Company).

### Density functional theory (DFT) calculations

DFT was used to calculate the electronic and piezoelectric properties of the amino acids using the plane wave-based Vienna Ab initio Simulation Package (VASP). The generalized gradient approximation was employed for the exchange−correlation function. An energy cutoff of 550 eV was used for the plane wave during geometry optimization, where the gamma-centered *k*-point meshes were 3 × 6 × 3, 2 × 6 × 3, and 4 × 6 × 2 for L-valine, L-leucine, and L-methionine amino acids, respectively. The corresponding 5 × 9 × 4, 3 × 9 × 5, and 5 × 10 × 3 *k*-point meshes were used for the piezoelectric properties’ calculations, respectively. The calculation was converged in energy to 10^−6^ eV per cell, and the structure was allowed to relax until the force was less than 2 × 10^−2^ eV/Å. Piezoelectric constant tensors (*d*_ik_) were obtained by converting from piezoelectric stress tensor (*e*_ij_) using the constitutive relation *e*_ij_ = *d*_ik_*C, where *C* presents the total elastic moduli.

DFT calculations were employed to investigate the HOMO and lowest unoccupied molecular orbital (LUMO) based on the Gaussian 09 package [36]. The dimer structures for calculation were derived from the single-crystal structures. All the structures were fully optimized at the B3LYP/6-31g(d,p) level of theory. The HOMO–LUMO gaps were obtained by the difference between the HOMO and LUMO orbitals. Orbital analyses are carried out using the multifunctional wavefunction analyzer (Multiwfn) software [37]. The visualization of the HOMO and LUMO orbitals is achieved through the Visual Molecular Dynamics (VMD) program [38].

### Piezoelectric force microscopy (PFM) measurement

PFM measurements of methionine crystals were carried out using a Cypher AFM (USA) in the dual AC resonance tracking (DART) mode. A conductive AFM probe, with a Pt/Ir coating film and a force constant of 2.8 N m^−1^ was used to scan over the surface of the sample along. AC stimulating voltages ranging from 0.5 to 5 V applied to the AFM probe cause the deformation of samples due to the piezoelectric effect, and the linear relationship between the change of the probe amplitude and the stimulating voltage can be used to calculate the effective piezoelectric coefficient. The in situ high-temperature piezoelectric response was measured at a temperature between 50 and 125 °C and controlled by a heating system of the AFM, and the annealing sample was measured at room temperature after high-temperature annealing treatments. All samples were measured with negligible surface electrostatic charges.

### Fabrication of nanogenerators based on methionine and valine crystals

The device was assembled as previously described [7]. Briefly, 2 Si substrates were cut into 1-cm × 1-cm squares, and two 0.7-cm × 0.7-cm Ag films were deposited on them as electrodes. Intermediate protective layers composed of Kapton tape, PDMS film, and double-side tapes were costed on the bottom substrate. A 0.7-cm × 0.7-cm hole was cut through the protected layer to expose the Ag electrode. Amino acid crystals were filled in the hole as a piezoelectric active layer, and a Si substrate with an Ag electrode was closely fixed on top of the layer. Two Cu wires connected the electrodes to complete the device. Finally, a Kapton tape was used to seal the device.

### Characterization of the power generator

The device was fixed perpendicular to a steel platform. A linear motor (E1100-RS-HC type with Force Control, LinMot) was employed to apply cyclic forces for the generation of electric signals. During the testing, the friction signal and Faradic currents might be created on the surface of the device, and these disturbing signals were largely prevented by closely pasting a conductive film over the device and the whole steel platform. The electrical output was measured with a low-noise voltage preamplifier (Stanford SR560) and recorded with a program written by LabVIEW. The device was contained in a Faraday cage, and all measurements were conducted in a copper screen room, such that electromagnetic noises and environmental disturbance were minimized.

## Data Availability

Data will be made available on request.

## Supplementary Materials

Supplementary Materials The supporting optical microscope images, XRD pattern, crystal structures, band structures and DOS, piezoelectric properties, and performance of nanogenerators are provided in the Supplementary Materials. Figs. S1 to S22 Table S1 and S2
