# Supplementary material for: Rational Design of Biological Crystals with Enhanced Physical Properties by Hydrogen Bonding Interactions
Source: Research (Wash D C). 2023 Feb 24;6:0046. doi: 10.34133/research.0046 (PMC10013789; doi:10.34133/research.0046)
Supplement: Supplementary Materials — The supporting optical microscope images, XRD pattern, crystal structures, band structures and DOS, piezoelectric properties, and performance of nanogenerators are provided in the Supplementary Materials. Figs. S1 to S22 Table S1 and S2 [file research.0046.f1.docx]

**Supporting Information**

**Rational design of biological crystals with enhanced physical properties by hydrogen bonding interactions**

Hui Yuan,^1,2^, Bin Xue,^3^ Dingyi Yang,^1^ Sigal Rencus-Lazar,^2^ Yi Cao,^3^ Ehud Gazit, ^*,1,2^ Dan Tan, ^*,1^ Rusen Yang^*,1^

^1^ School of Advanced Materials and Nanotechnology, Xidian University, Xi’an 710126, China

^2^ The Shmunis School of Biomedicine and Cancer Research, George S. Wise Faculty of Life Sciences; Department of Materials Science and Engineering, The Iby and Aladar Fleischman Faculty of Engineering, Tel Aviv University, Tel Aviv 6997801, Israel

^3^ National Laboratory of Solid State Microstructure, Department of Physics, Nanjing University, Nanjing 210093, Jiangsu, China

E-mail: [rsyang@xidian.edu.cn](mailto:rsyang@xidian.edu.cn) (R. Y.), [ehudg@post.tau.ac.il](mailto:ehudg@post.tau.ac.il) (E. G.), [dtan@xidian.edu.cn](mailto:dtan@xidian.edu.cn) (D. T.)


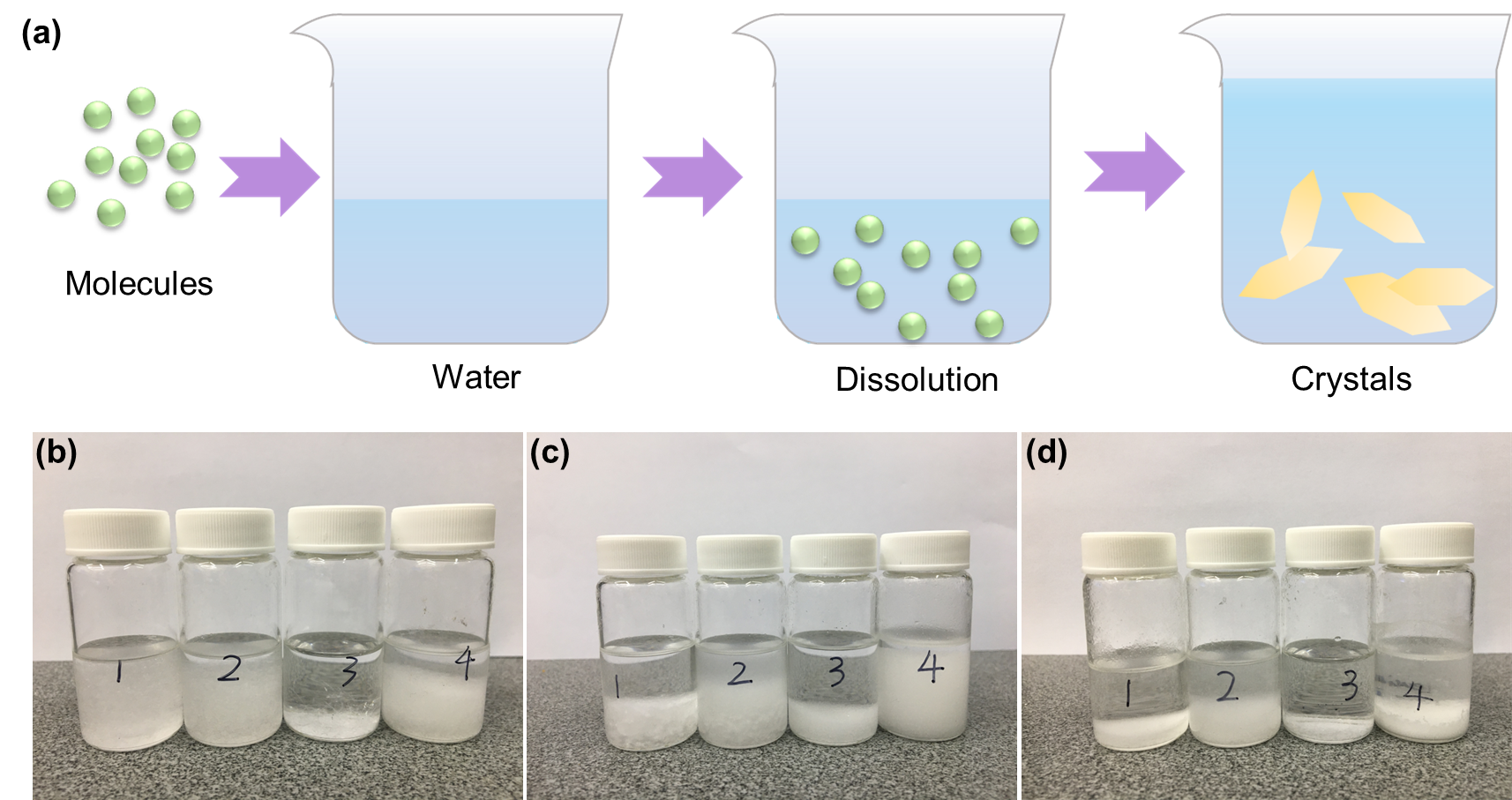


Figure S1 (a) A growth diagram of valine, leucine, and methionine crystals in water and organic solvent mixed solutions. (b-d) photographs of (b) valine, (c) leucine, and (d) methionine grown in various solutions. (1) Deionized water and ethanol mixed solution. (2) Deionized water and isopropanol mixed solution. (3) Deionized water and MeOH mixed solution. (4) Deionized water and acetonitrile mixed solution.


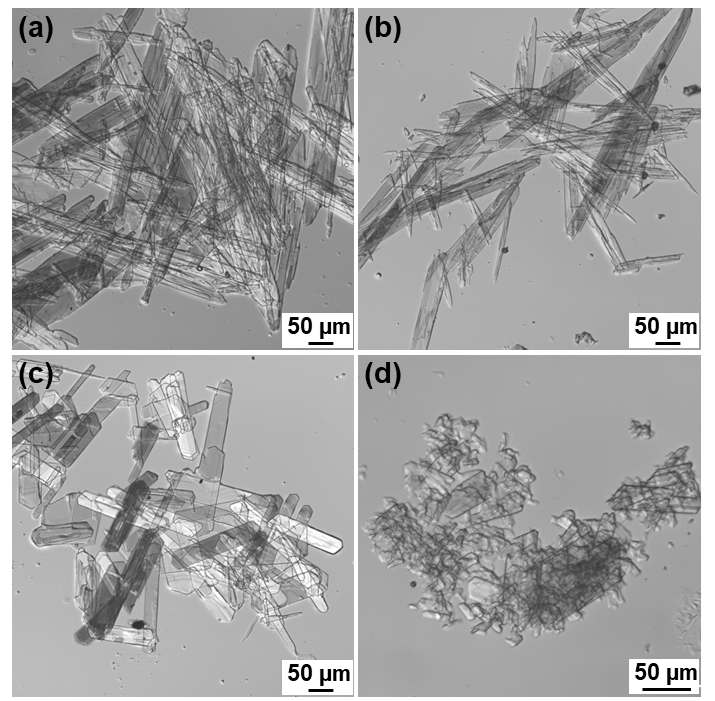


Figure S2 Optical microscope images of valine grown in (a) deionized water and ethanol mixed solution, (b) deionized water and isopropanol mixed solution, (c) deionized water and MeOH solution, and (d) deionized water and acetonitrile solution. Scare bar: 50 µm.


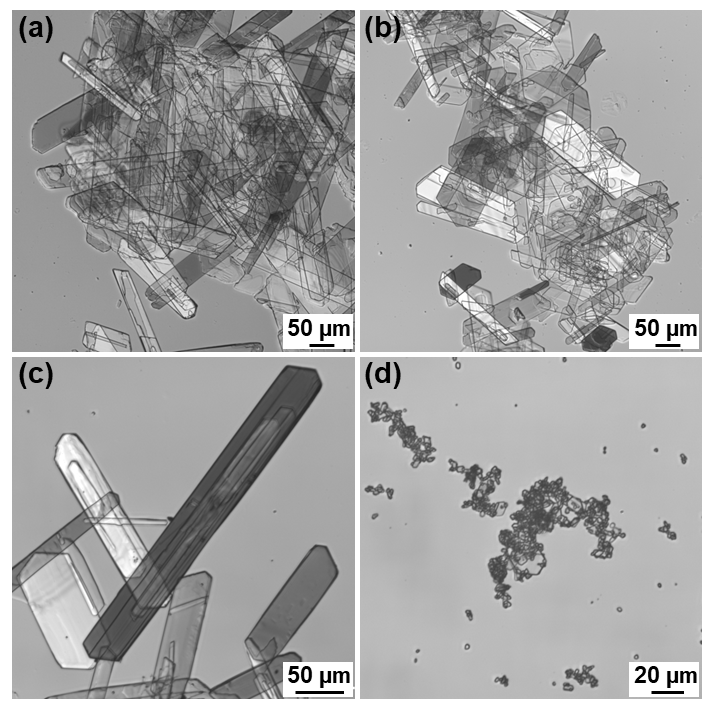


Figure S3 Optical microscope images of leucine grown in (a) deionized water and ethanol mixed solution, (b) deionized water and isopropanol mixed solution, (c) deionized water and MeOH solution, and (d) deionized water and acetonitrile solution. Scale bar (a-c): 50 µm, (d): 20 µm.


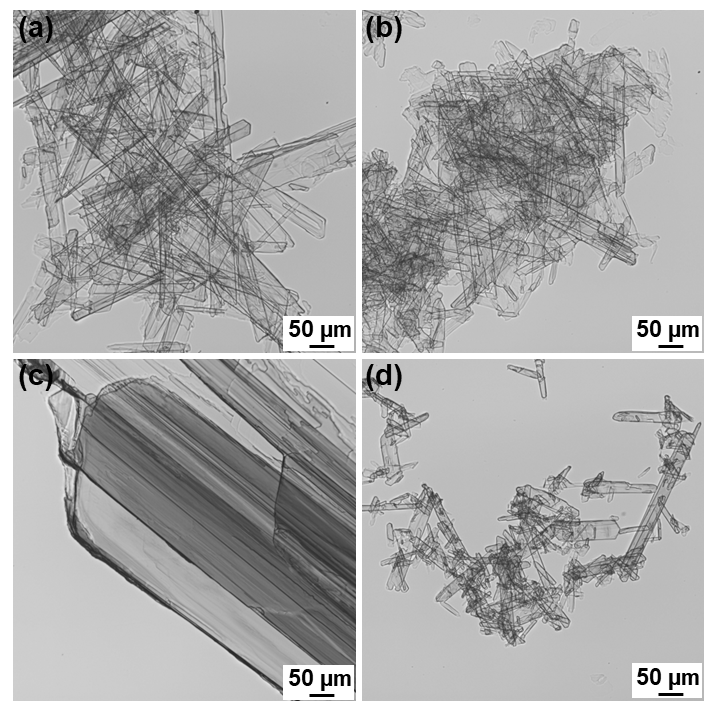


Figure S4 Optical microscope images of methionine grown in (a) deionized water and ethanol mixed solution, (b) deionized water and isopropanol mixed solution, (c) deionized water and MeOH solution, and (d) deionized water and acetonitrile solution. Scale bar: 50 µm.

Figure S5 XRD patterns of valine sheets grown in water and organic solvent mixtures, as indicated.

Figure S6 XRD patterns of leucine sheets grown in water and organic solvent mixtures, as indicated.


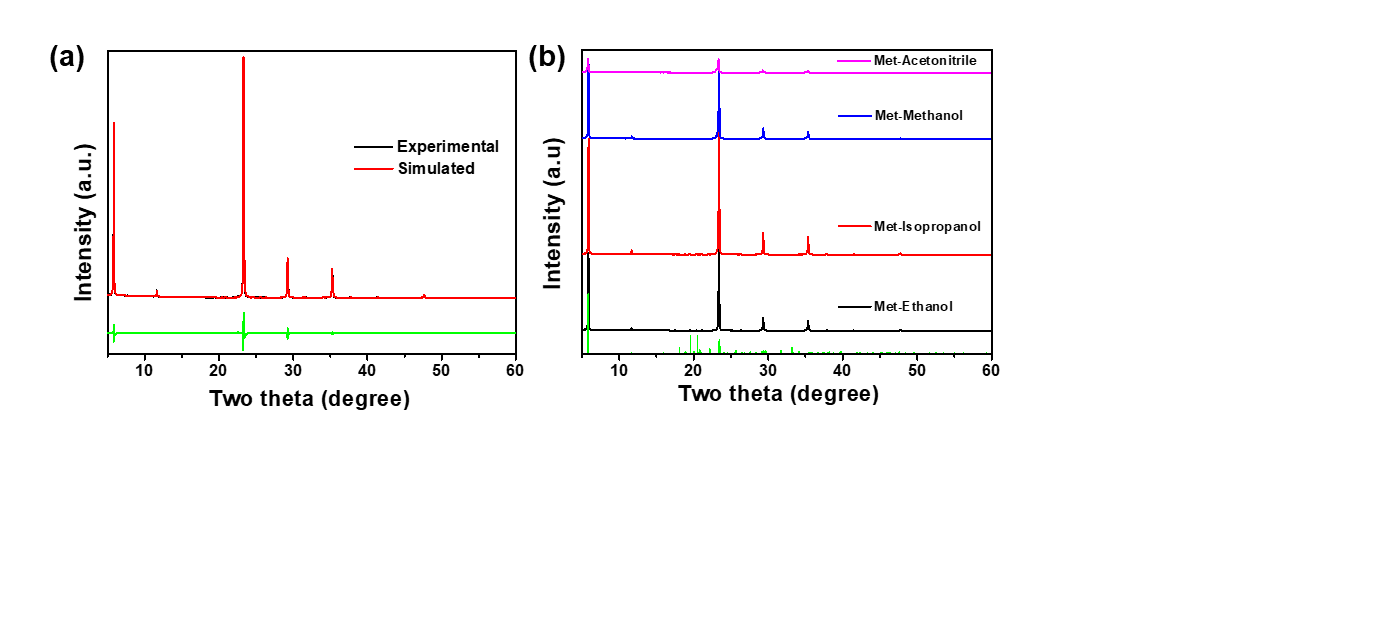


Figure S7 XRD patterns of methionine sheets (a) matched with the simulated result, (b) grown in water and organic solvent mixtures, as indicated.


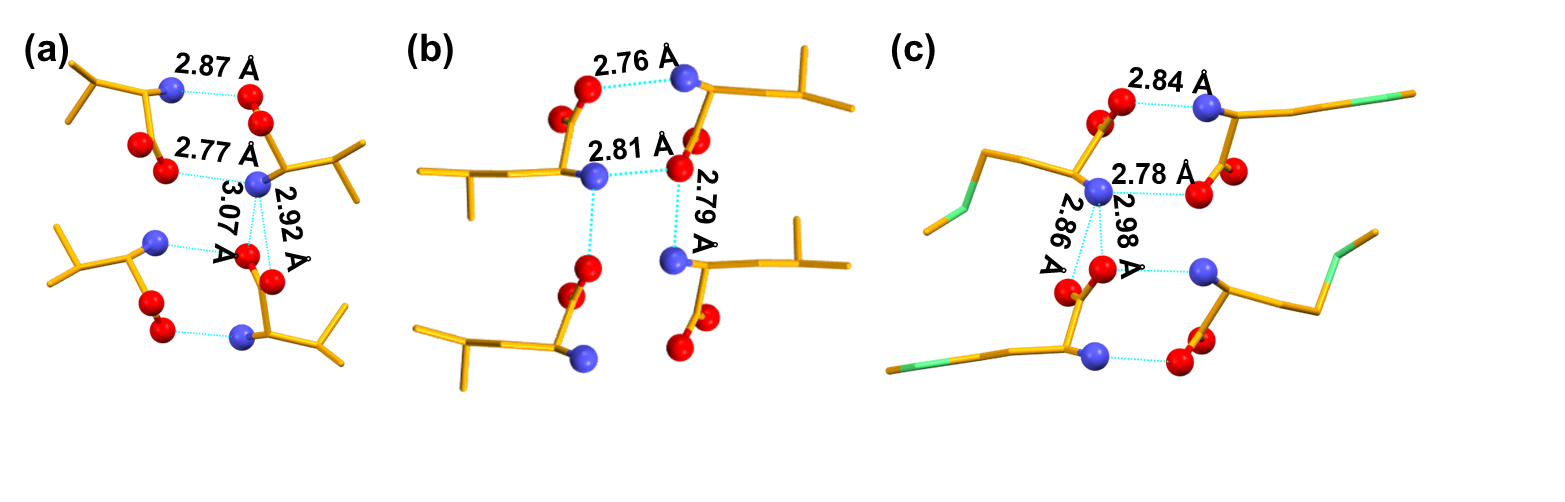


Figure S8 The hydrogen-bonding network of (a) valine, (b) leucine, and (c) methionine crystals. Color code: orange, C; blue, N; red, O; light green, S; and wathet broken line, hydrogen bonding.


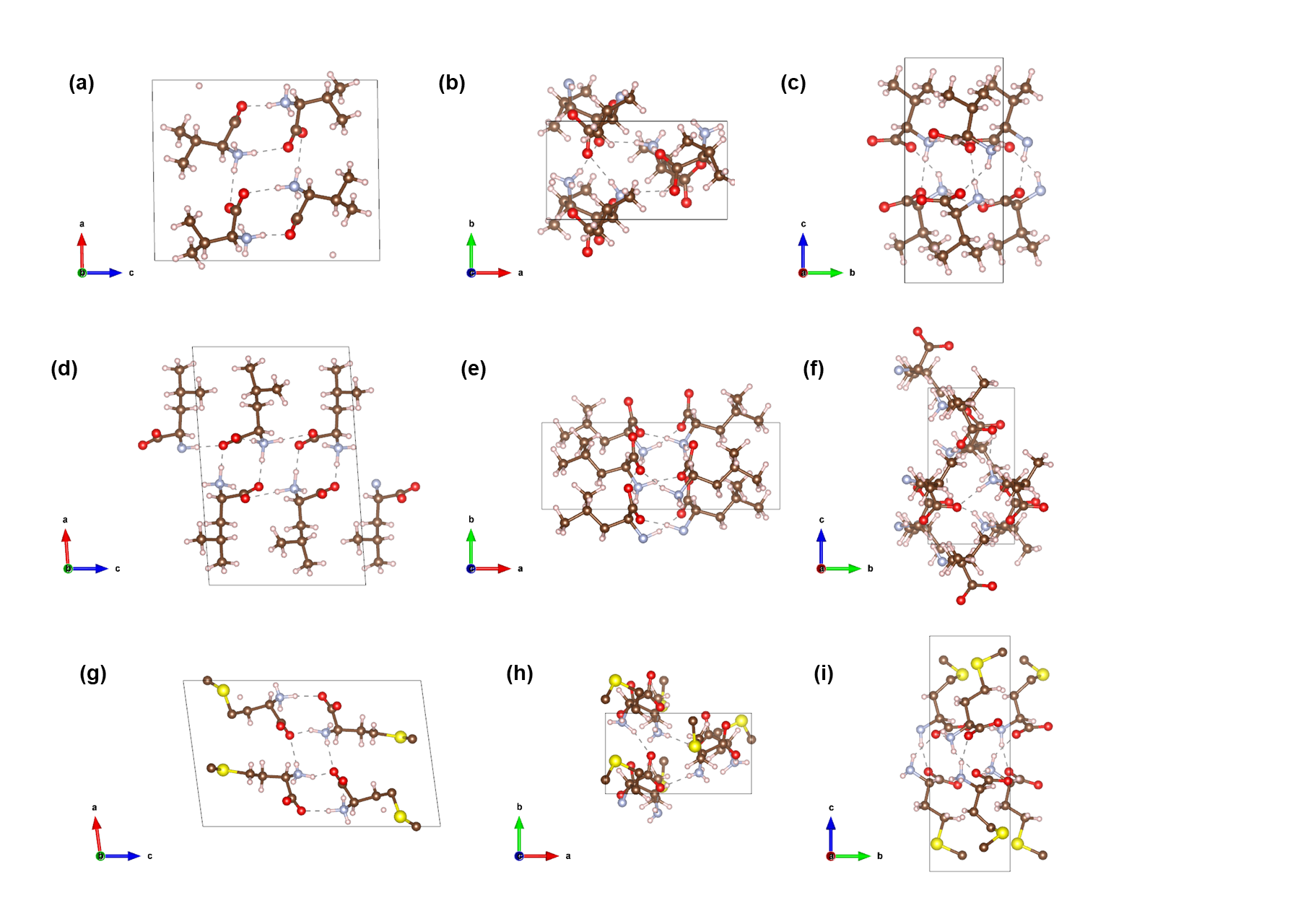


Figure S9 Calculated crystal structure visualized along various directions of (a-c) valine, (d-f) leucine, and (g-i) methionine for electric band structures and density of states properties.


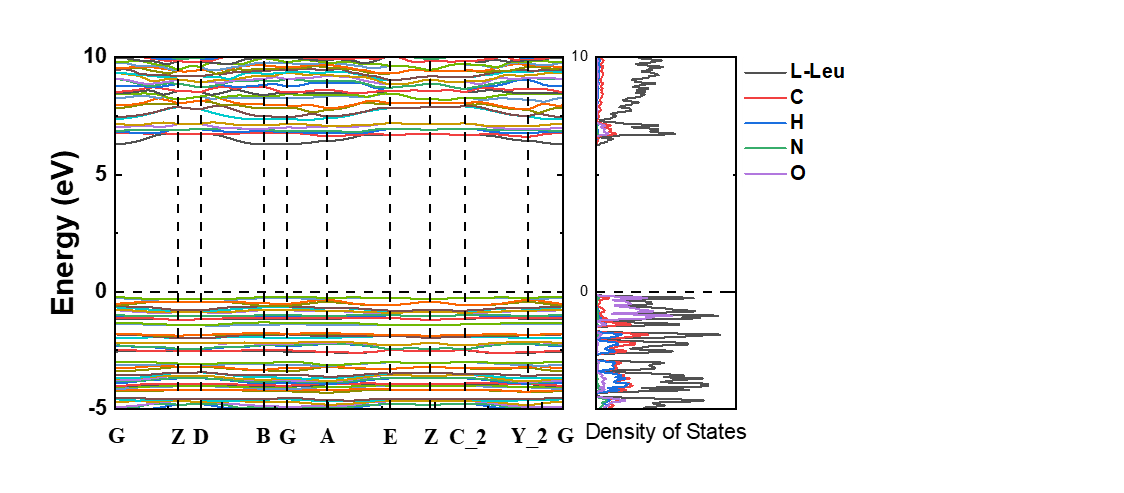


Figure S10 Band structures and density of states of leucine crystals.


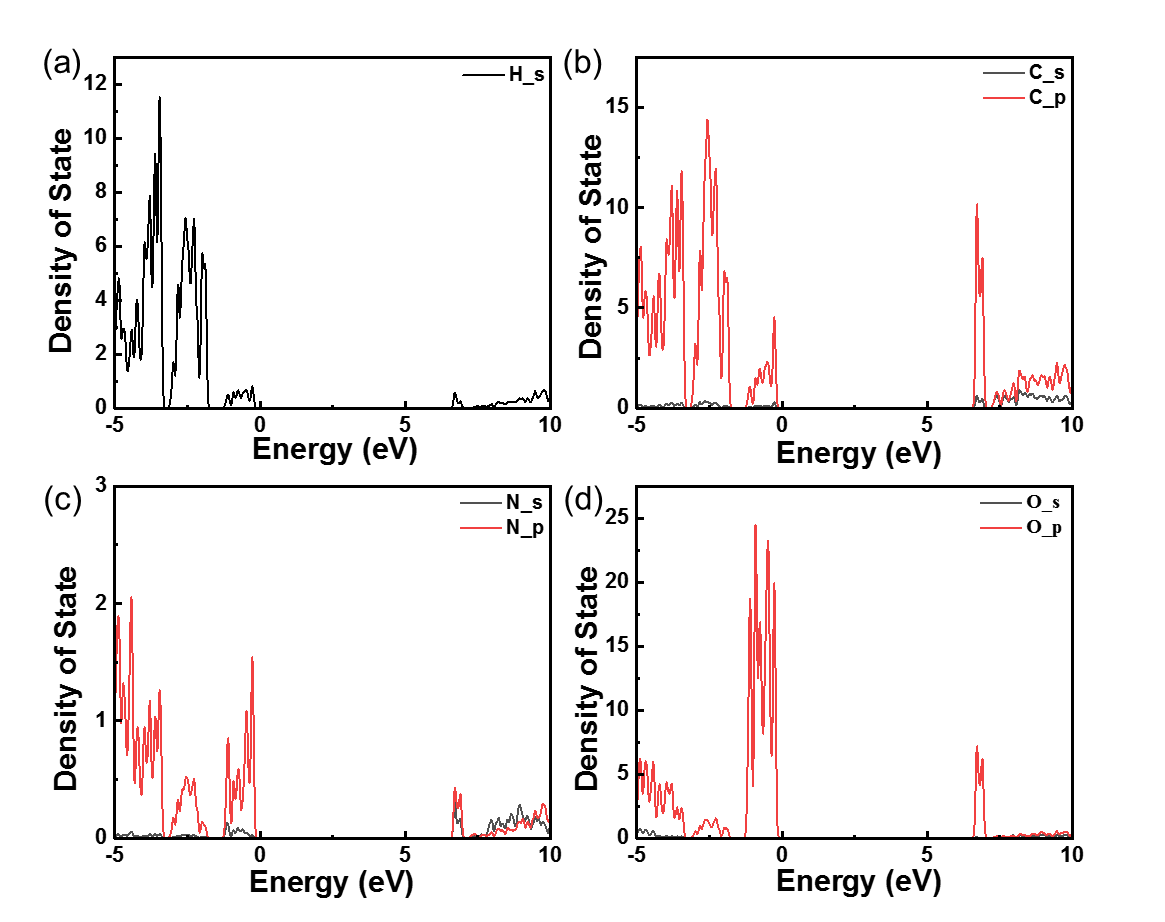


Figure S11 PDOS of valine crystals for (a) H, (b) C, (c) N, and (d) O.


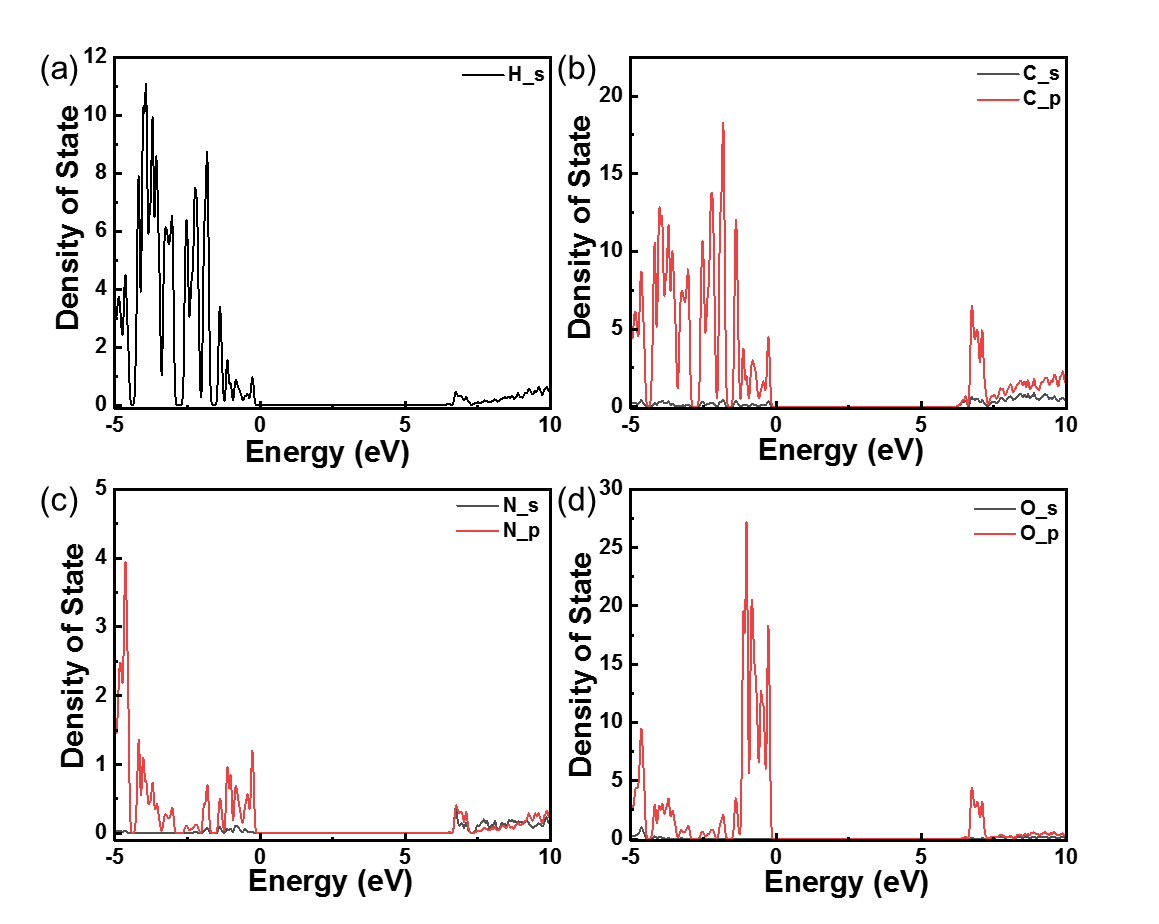


Figure S12 PDOS of leucine crystals for (a) H, (b) C, (c) N, and (d) O.


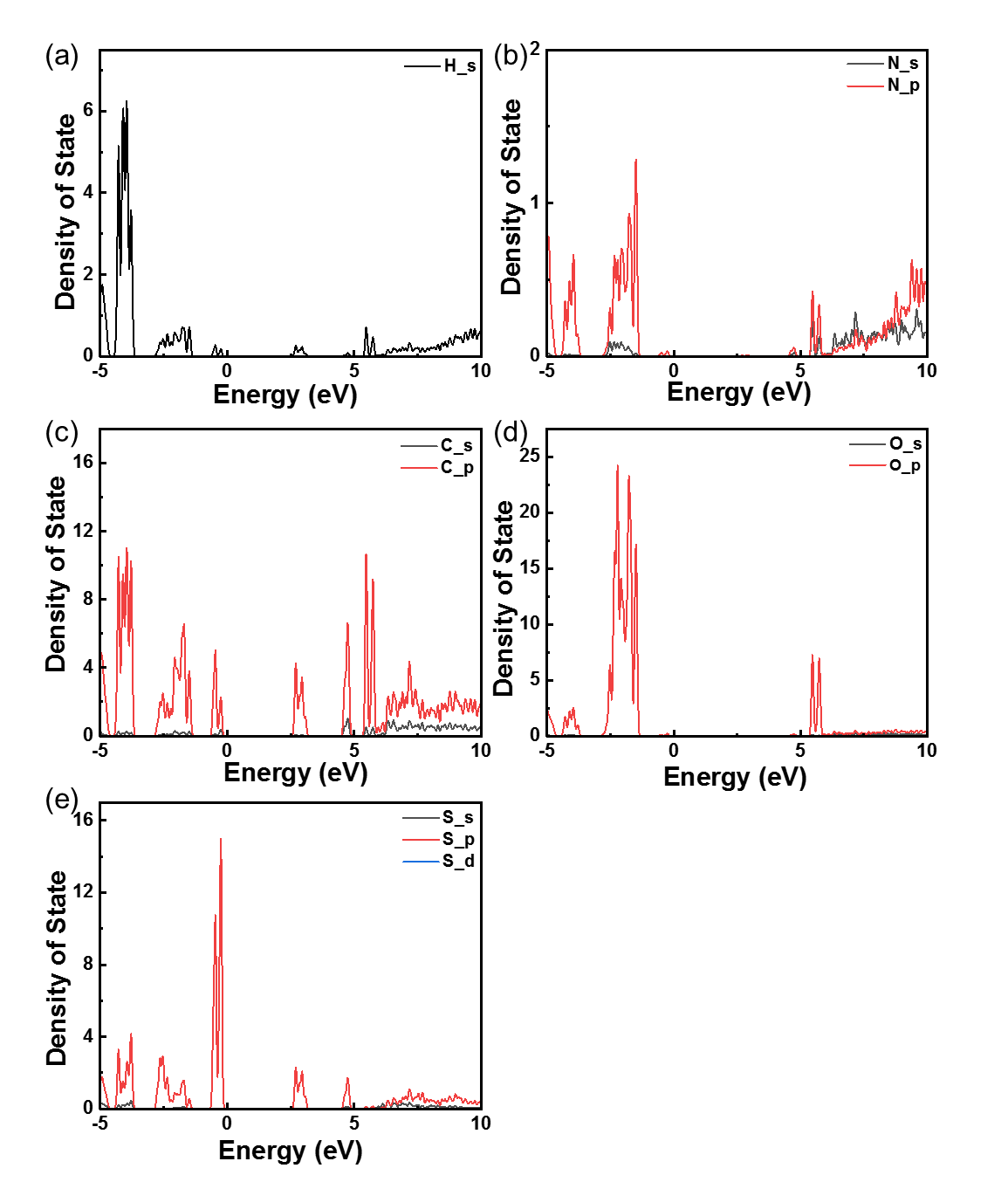


Figure S13 PDOS of methionine crystals for (a) H, (b) C, (c) N, (d) O, and (e) S.


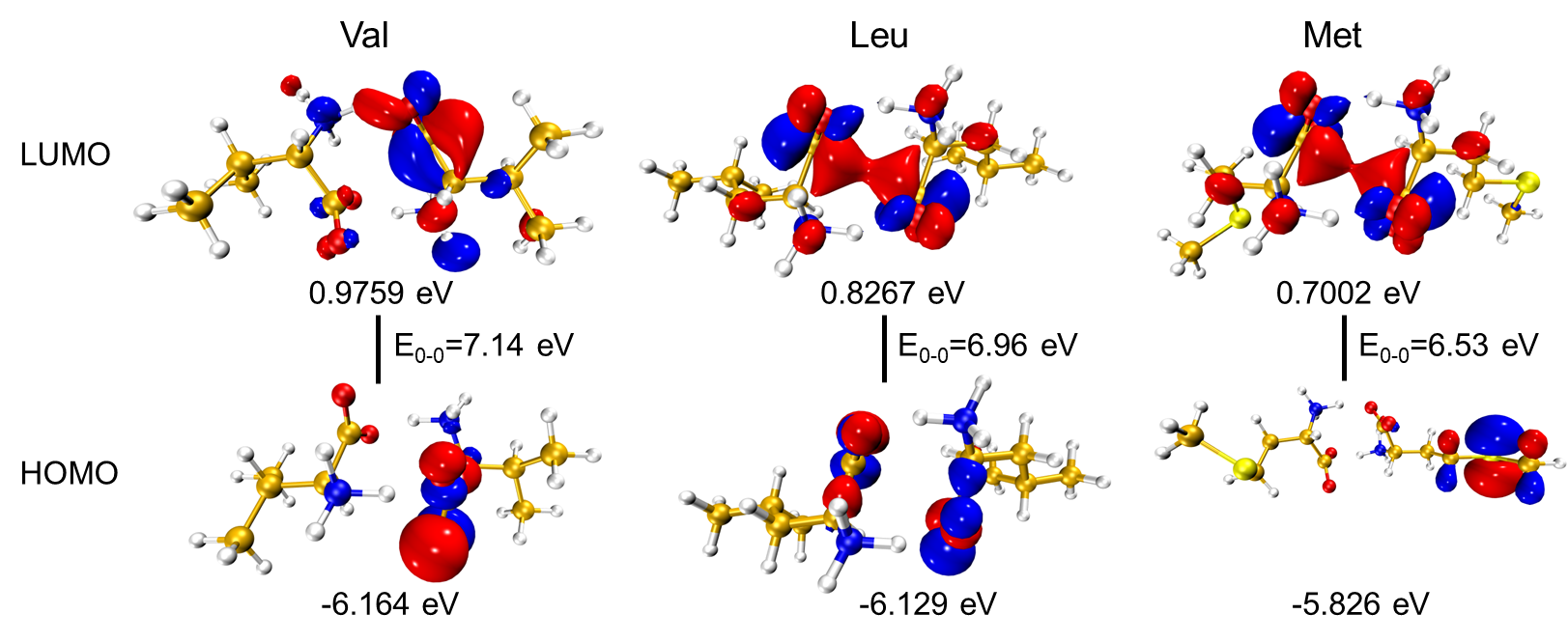


Figure S14 HOMO and LUMO molecular orbitals of valine, leucine, and methionine dimers, demonstrating the conductance gaps of 7.14, -6.96 and -6.53 eV, respectively.


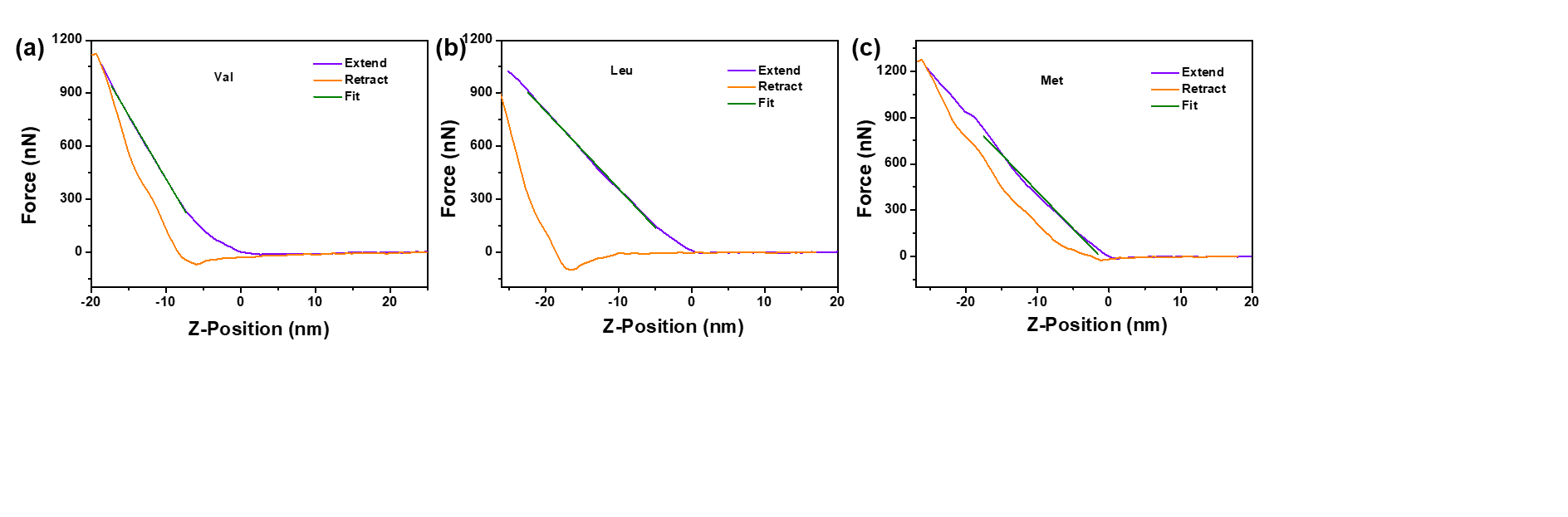


Figure S15 Typical force-distance traces of (a) valine, (b) leucine, and (c) methionine crystals. The olive line demonstrates the fitting result from the “extend” trace using the Hertz model.


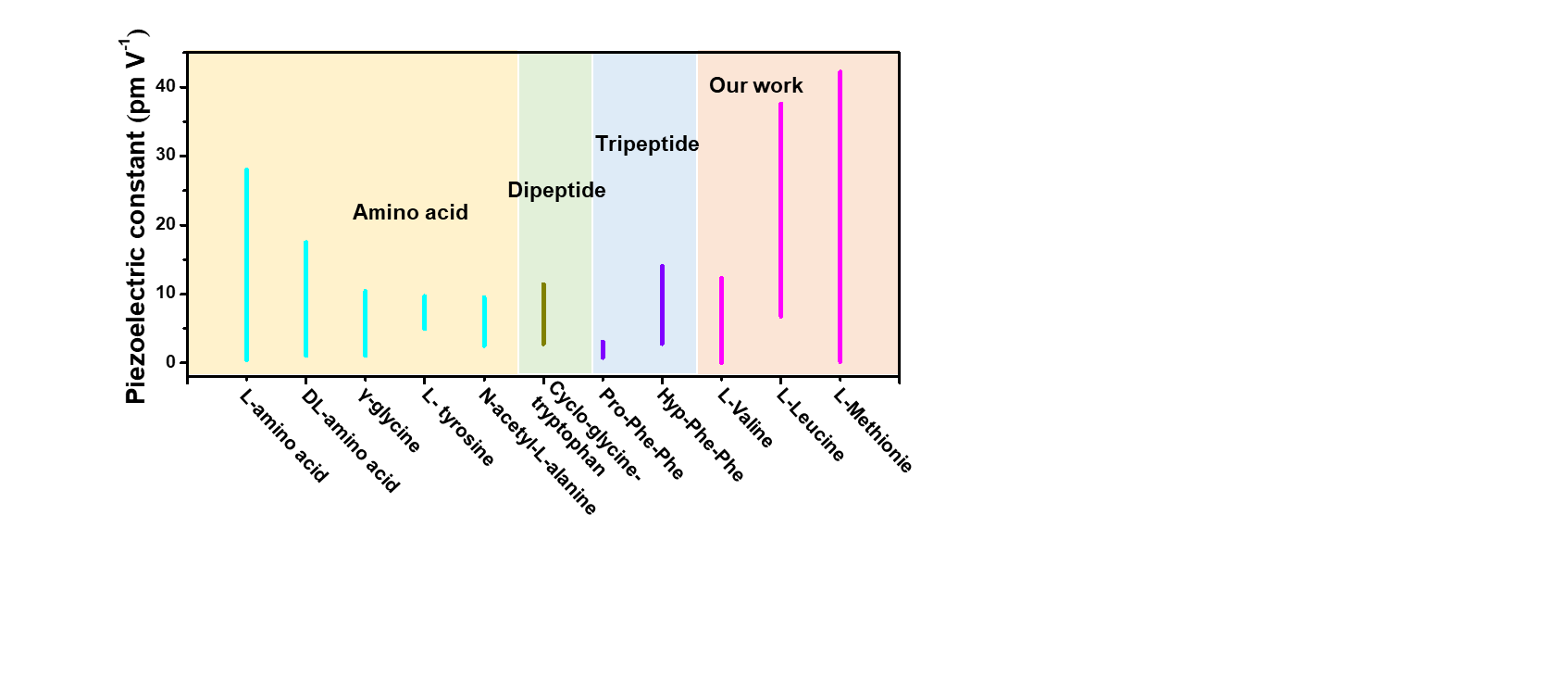


Figure S16 The piezoelectric properties of amino acid crystals analyzed in this work compared with other biomaterials.^1-6^

Figure S17 XRD spectra of methionine crystals before and after annealing at 150 ℃ for 0.5 h.


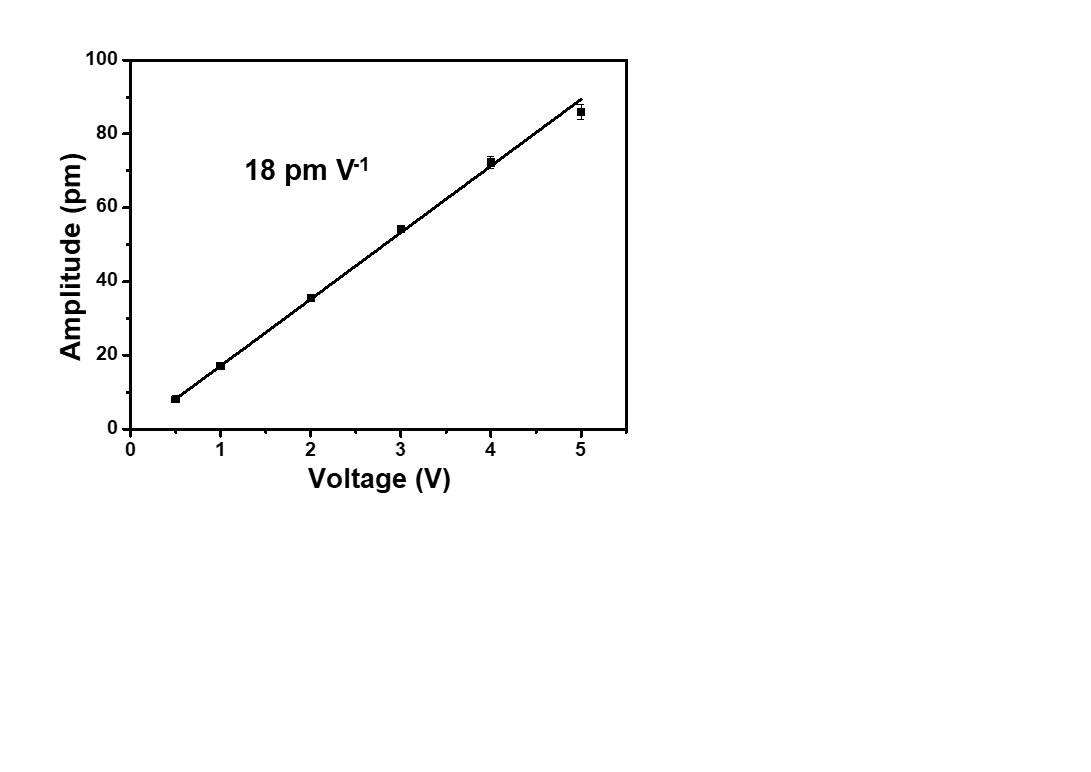


Figure S18 Tested effective piezoelectric constant of methionine crystals along the thickness direction.


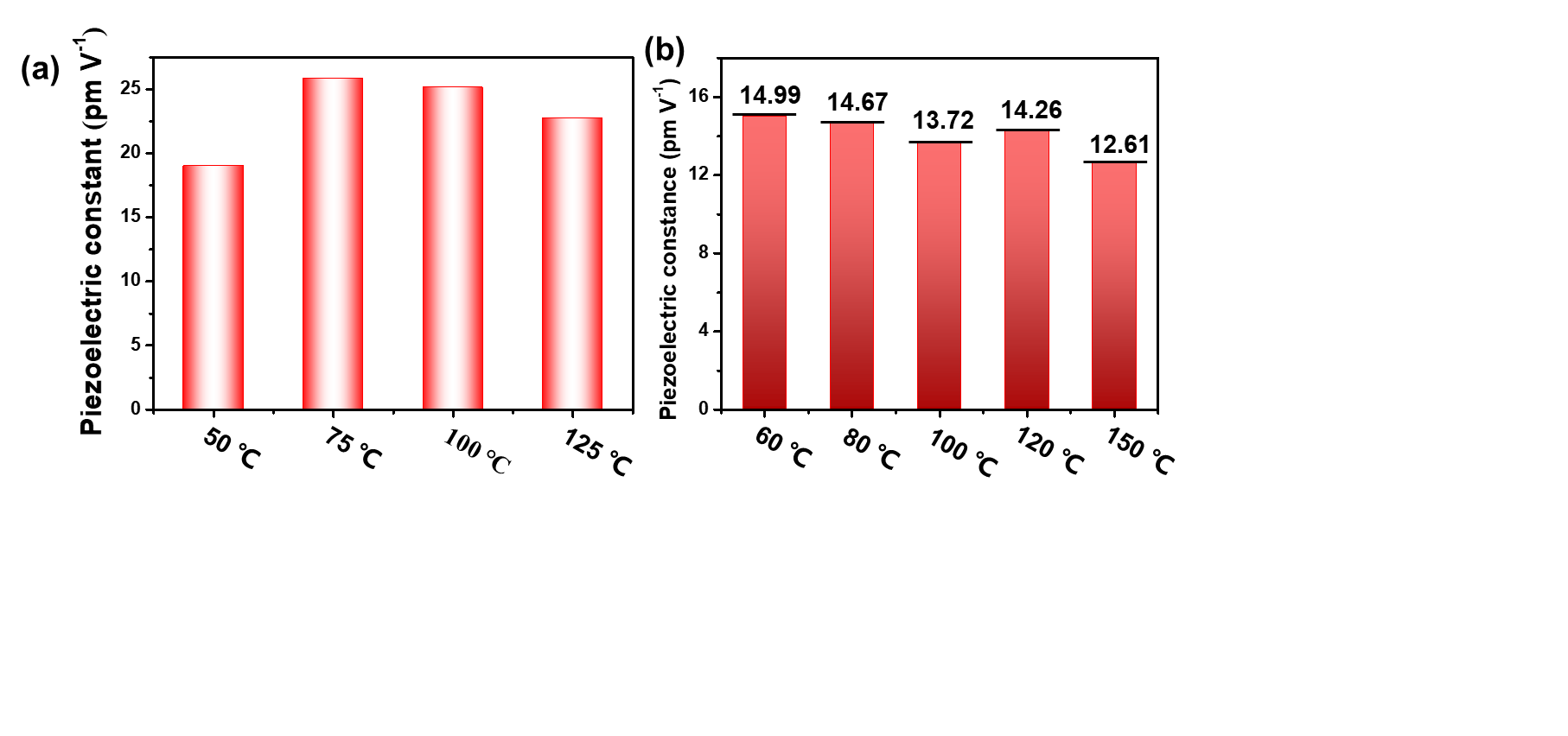


Figure S19 Tested effective piezoelectric constants of methionine crystals (a) with in situ high-temperature tests, and (b) after annealing at different temperatures for 0.5 h along the thickness direction.


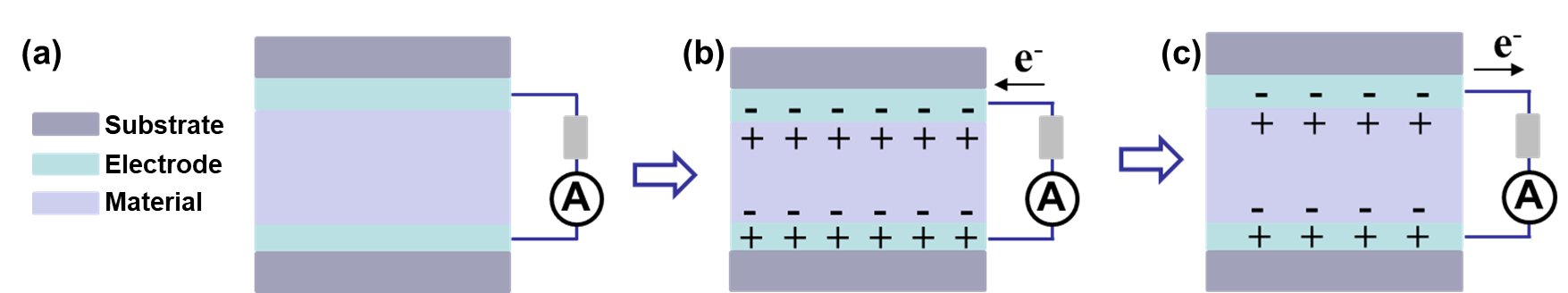


Figure S20 The operating mechanism of the biomaterials-based nanogenerators. (a) No polarization produces without applied force. (b) Polarization charges generate on the surface of piezoelectric crystals under applied force drive the flow of electrons between two electrodes. (c) Electrons flow back rapidly upon the force is canceled.


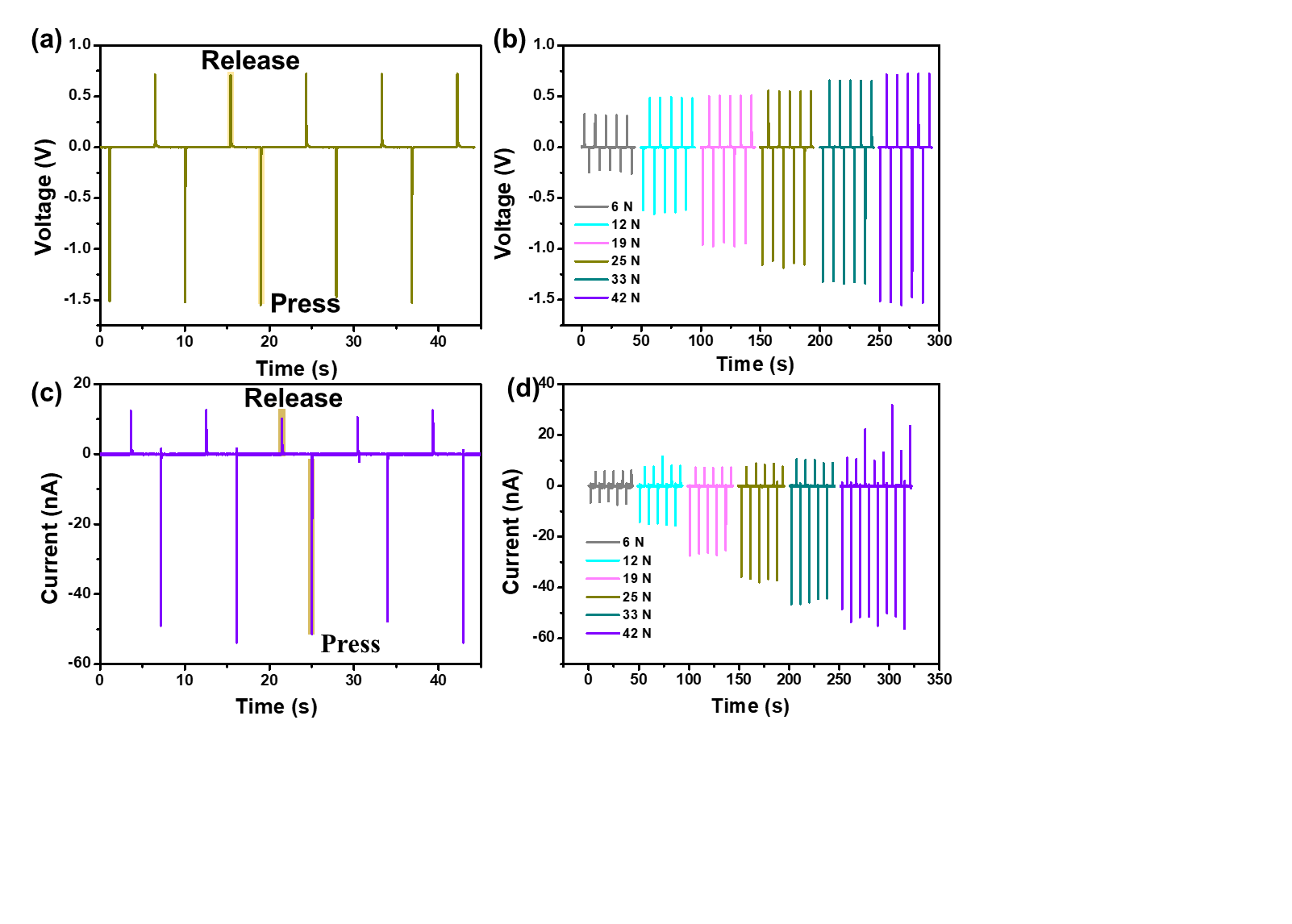


Figure S21 (a, b) Output voltage produced by methionine-based nanogenerators (a) under applied a force of 42 N and (b) under various forces in the reversed connection. Output current produced by methionine-based nanogenerators (c) under an applied force of 42 N and (d) under various forces in the reversed connection.


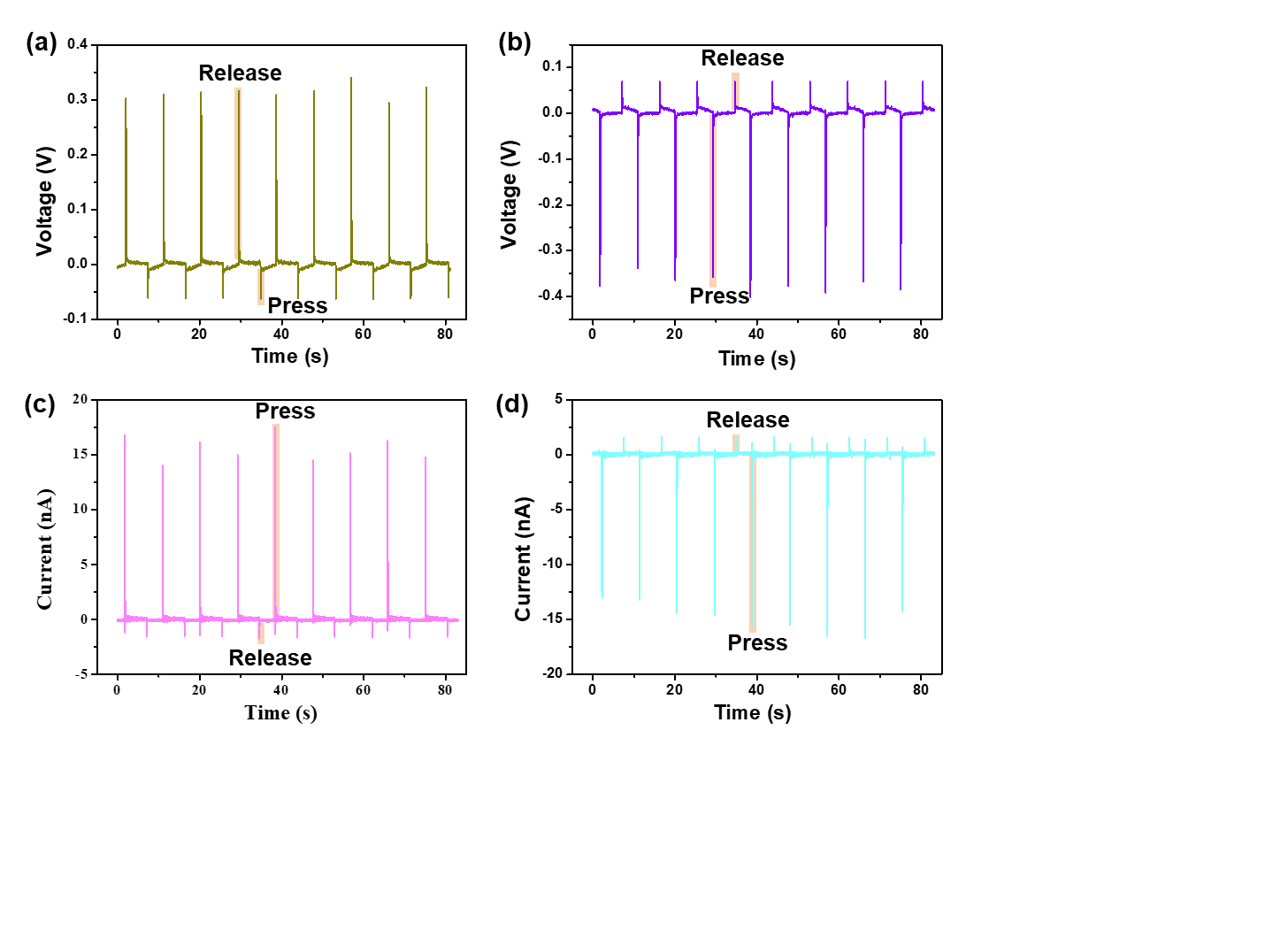


Figure S22 (a) Open-circuit voltage produced by valine-based nanogenerators under an applied force of 42 N and (b) in the reversed connection. (c) Short-circuit current produced by valine-based nanogenerators under an applied force of 42 N and (d) in the reversed connection.

**Table S1** Calculated piezoelectric charge tensor components e_ij_ (in units of C m^-2^), Total Elastic Moduli (kBar), and strain tensor components d_ik_ (pm V^-1^) of leucine crystals.

**Charge Tensor (C m^-2^)**

$$\left( \begin{matrix} \text{0} & \text{0} & \text{0} \\ \text{-0.1389} & \text{-0.3304} & \text{ 0.0226} \\ \text{0} & \text{0} & \text{0} \end{matrix}\text{ }\begin{matrix} \text{-0.0078} & \text{0} & \text{0.0399} \\ \text{0} & \text{-0.0446} & \text{ 0} \\ \text{-0.0156} & \text{0} & \text{0.0736} \end{matrix} \right)$$

**Total Elastic Moduli (kBar)**

$$\left( \begin{aligned} \begin{matrix} \text{395.2558} & \text{128.1912} & \text{24.3681} \\ \text{128.1912} & \text{278.4622} & \text{20.8372} \\ \text{24.3681} & \text{20.8372} & \text{80.7147} \end{matrix}\text{ }\begin{matrix} \text{ 0} & \text{ 2.19920 } & \text{0} \\ \text{ 0} & \text{ -0.1967} & \text{0} \\ \text{ 0} & \text{ -4.8623} & \text{0} \end{matrix} \\ \\ \begin{matrix} \text{ 0} & \text{ 0} & \text{ 0} \\ \text{ 0} & \text{ 0} & \text{ 0} \\ \text{ 2.1992} & \text{-0.1967} & \text{-4.8623} \end{matrix}\text{ }\begin{matrix} \text{ -8.1085} & \text{ 0} & \text{ 96.0871} \\ \text{ 33.8935} & \text{-8.1085} & \text{0} \\ \text{0} & \text{59.5764} & \text{0} \end{matrix} \end{aligned} \right)$$

**Strain Tensor (pm V^-1^)**

$$\left( \begin{matrix} \text{0} & \text{0} & \text{0} \\ \text{0.24413} & \text{13.8348} & \text{ -10.1111} \\ \text{0} & \text{0} & \text{0} \end{matrix}\text{ }\begin{matrix} \text{-7.664} & \text{0} & \text{22.9178} \\ \text{0 } & \text{-23.2916} & \text{ 0} \\ \text{-14.3565} & \text{0} & \text{42.4601} \end{matrix} \right)$$

**Table S2** Calculated piezoelectric charge tensor components e_ij_ (in units of C m^-2^), Total Elastic Moduli (kBar), and strain tensor components d_ik_ (pm V^-1^) of methionine crystals

**Charge Tensor (C m^-2^)**

$$\left( \begin{matrix} \text{0} & \text{0} & \text{0} \\ \text{0.0247} & \text{0.2999} & \text{ -0.1998} \\ \text{0} & \text{0} & \text{0} \end{matrix}\text{ }\begin{matrix} \text{-0.0262} & \text{0} & \text{-0.0883} \\ \text{0} & \text{0.1598} & \text{ 0} \\ \text{-0.0687} & \text{0} & \text{0.0702} \end{matrix} \right)$$

**Total Elastic Moduli (kBar)**

$$\left( \begin{aligned} \begin{matrix} \text{344.6743} & \text{149.5736} & \text{149.5736} \\ \text{149.5736} & \text{152.5539} & \text{75.3555} \\ \text{108.3728} & \text{108.3728} & \text{333.6764} \end{matrix}\text{ }\begin{matrix} \text{ 0} & \text{ 2.3668 } & \text{0} \\ \text{ 0} & \text{ -7.3545} & \text{0} \\ \text{ 0} & \text{ -24.3586} & \text{0} \end{matrix} \\ \\ \begin{matrix} \text{ 0} & \text{ 0} & \text{ 0} \\ \text{ 0} & \text{ 0} & \text{ 0} \\ \text{ 2.3668} & \text{-7.3545} & \text{-24.3586} \end{matrix}\text{ }\begin{matrix} \text{ -24.3586} & \text{ 2.3630} & \text{0} \\ \text{ 2.3630} & \text{-43.6172} & \text{0} \\ \text{0} & \text{0} & \text{92.4063} \end{matrix} \end{aligned} \right)$$

**Strain Tensor (pm V^-1^)**

$$\left( \begin{matrix} \text{0} & \text{0} & \text{0} \\ \text{-12.922} & \text{ 37.634} & \text{ -8.958} \\ \text{0} & \text{0} & \text{0} \end{matrix}\text{ }\begin{matrix} \text{6.825} & \text{0} & \text{15.016} \\ \text{0 } & 18.262 & \text{ 0} \\ \text{16.374} & \text{0} & \text{11.500} \end{matrix} \right)$$

**References**

1. S. Guerin, A. Stapleton, D. Chovan, et al., "Control of Piezoelectricity in Amino Acids by Supramolecular Packing" *Nature Materials*, vol 17, pp. 180-186, 2018.

2. S. Bera, S. Guerin, H. Yuan, et al., "Molecular Engineering of Piezoelectricity in Collagen-Mimicking Peptide Assemblies" *Nature communications*, vol 12, pp. 1-12, 2021.

3. S. Guerin, S. A. M. Tofail, and D. Thompson, "Longitudinal Piezoelectricity in Orthorhombic Amino Acid Crystal Films" *Crystal Growth & Design*, vol 18, pp. 4844-4848, 2018.

4. S. Guerin, J. O'Donnell, E. U. Haq, et al., "Racemic Amino Acid Piezoelectric Transducer" *Physical Review Letters*, vol 122, pp. 047701, 2019.

5. W. Ji, B. Xue, S. Bera, et al., "Tunable Mechanical and Optoelectronic Properties of Organic Cocrystals by Unexpected Stacking Transformation from H-to J-and X-Aggregation" *ACS nano*, vol 14, pp. 10704-10715, 2020.

6. K. Tao, W. Hu, B. Xue, et al., "Bioinspired Stable and Photoluminescent Assemblies for Power Generation" *Advanced Materials*, vol 31, pp. 1807481, 2019.
